# Effects of Sub-Chronic Exposure to Polystyrene Nanoplastics on Lipid and Antioxidant Metabolism in *Sparus aurata*

**DOI:** 10.3390/ani15040562

**Published:** 2025-02-14

**Authors:** Ekemini Okon, Irene Brandts, Hayam Djafar, Asta Tvarijonaviciute, Joan Carles Balasch, Mariana Teles

**Affiliations:** 1 Department of Cell Biology, Physiology and Immunology, Universitat Autònoma de Barcelona, 08193 Barcelona, Spain; okon.ekeminimoses@gmail.com (E.O.); irene.brandts@uab.cat (I.B.); hayamdj30@gmail.com (H.D.); 2 Interdisciplinary Laboratory of Clinical Analysis INTERLAB-UMU, Regional Campus of International Excellence Mare Nostrum, University of Murcia, Espinardo, 30100 Murcia, Spain; asta@um.es

**Keywords:** nanoplastics, lipid metabolism, antioxidant response, liver, histopathology, hematology, *Sparus aurata*

## Abstract

Nanoplastics (NPs) can cross cellular membranes and affect cellular performance. This study aims to determine the effects of polystyrene NPs (PS-NPs, 44 nm) on gilthead seabream (*Sparus aurata*) exposed for 14 days to 100 μg/L PS-NPs. The results show that biometric indicators (weight, length, Fulton’s condition factor, and hepatosomatic index) were not affected after the experimental exposures. No significant effects were observed on white blood cell count, red blood cell count, mean corpuscular hemoglobin, or platelets compared to the control group. However, there was a significant decrease in hemoglobin concentration, hematocrit values, and mean corpuscular cell volume in fish exposed to PS-NPs. There were no significant effects on plasmatic cholesterol, triglyceride, alkaline phosphatase, or aspartate aminotransferase levels. The histological anatomy of both the gills and the intestine revealed no obvious signs of cellular damage, excessive mucous, or inflammation in the PS-NP group. The expression of transcripts related to lipid metabolism (*pparα*, *pparβ*), growth and development (*igf1*), detoxification (*bche*), and oxidative stress (*sod*, *gpx1*) were significantly downregulated in animals exposed to PS-NPs, indicating a slight impairment in lipid homeostasis and antioxidant response.

## Simple Summary

Plastic waste constitutes a significant threat to the health of aquatic animals. Due to their small size, high surface profile, high mobility, and pervasiveness, nanoplastics (NPs) have colonized all terrestrial and aquatic environments. NPs can enter food chains, travel through the blood, cross cell membranes, and accumulate in tissues and organs. Several studies have focused on the short-term effects of NPs on the health of aquatic animals, but the medium-term or chronic effects are still obscure. Here, we have analyzed the putative detrimental effects of 14-day exposure to 100 μg/L of polystyrene nanoplastics (PS-NPs), one of the most abundant types of NPs found in aquatic ecosystems, on the physiology of gilthead seabream, an abundant commercial fish species in the Mediterranean Sea. After analyzing several biochemical, hematological, and genetic markers of altered metabolism in the liver and blood, together with changes in the structure of the gills and intestine, we found minor changes in the management of lipid metabolism, oxidative stress, and hematological parameters in fish exposed to PS-NPs, with no alterations in the morphology of the gills and intestine. If maintained, chronic exposure to PS-NPs may result in an accretive process of physiological disturbance.

## 1. Introduction

In marine ecosystems, the threat of plastic pollution is widespread and increasing, becoming a significant environmental concern in recent years and capturing global attention [1,2,3]. The widespread use of plastics has culminated in their extensive dispersal into the natural environment, including aquatic ecosystems. The discharge of plastics into the aquatic environment occurs through several channels, encompassing runoff from land and wastewater, uncontrolled littering, illegal dumping methods, and the prevalent use of plastics in maritime activities [4]. In 2019 alone, about 82–358 trillion plastic particles (weighing 1.1–4.9 million tonnes, Mt) ended up in the ocean [5]. Despite this challenge, plastic production is still increasing exponentially on a global scale and generating waste. Global production figures increased from 2 Mt in 1950 to approximately 400 Mt in 2019, of which 57.2 Mt was produced in Europe [6]. Furthermore, plastics are resistant to biodegradation and can persist in the environment for several decades or even centuries, degrading over time to microplastics and nanoplastics [7]. This plastic waste constitutes a significant threat to the health of aquatic animals, including fish, regardless of their size and nature.

Nanoplastics (NPs), particles measuring less than 1000 nm in diameter [8], have emerged as a concerning environmental pollutant because of their extensive presence in aquatic environments. However, the detection of NPs poses several challenges, as it has not yet been optimally refined in aquatic ecosystems [9,10], and therefore NPs are often excluded from oceanographic surveys. As a result, globally established environmental concentrations are lacking [11]. Nevertheless, studies in Mediterranean waters have established the presence of NPs [12], with concentrations similar to those found in oceanic regions. This is attributed to intensive aquaculture production in the area and the high input of plastic loads into the Mediterranean Sea from different sources [13,14,15]. Due to their nanoscale physicochemical properties, allowing them to cross biological barriers and cell membranes [16,17] and accumulate in organs [18], NPs potentially have more detrimental effects on aquatic organisms than micro- and macroplastics. Moreover, NPs can act as a means of transporting chemical and biological xenobiotics into aquatic organisms [19]. Therefore, they pose diverse threats to marine life, ranging from ingestion by marine organisms to facilitating the entry of pollutants into the marine trophic food chain [20,21]. Previous studies on the effects of NPs in fish have described bioaccumulation in the muscle and liver tissue [22], disruption of the immune system [23], oxidative stress and liver damage [24], and DNA damage and genotoxicity [22], among others. Given that in natural ecosystems, fish face a significant probability of exposure to NPs, these particles can be transferred up the food chain to higher trophic-level organisms [25,26,27].

The present study aimed to determine the potential detrimental effects of polystyrene nanoplastics (PS-NPs) at distinct biological levels in gilthead seabream (*Sparus aurata*), a commercially important fish relevant to both fisheries and aquaculture production [28]. A 14-day experimental duration was chosen as this aligns with established protocols for short- to medium-term exposure studies in fish, which typically range from 7 to 14 days [29]. The effects of these stressors were evaluated after both individual exposure and co-exposure for 14 days, using biomarkers encompassing different levels of biological organization, from the molecular (gene expression) to the whole-individual level (biochemical, hematological, and histological). This study will provide valuable information on the potential threats to the health and welfare of this commercially important fish species. Furthermore, this study could have broader implications for the management of other marine species that are exposed to these pressing environmental stressors beyond the Mediterranean regions.

## 2. Materials and Methods

### 2.1. Fish Maintenance

Gilthead seabream (*Sparus aurata*) juveniles, weighing approximately 8 g, were acquired from an aquaculture facility (AVRAMAR, Vall d’Uixó, Castellon, Spain) and transported to the UAB fish facility. They were then moved to a 1000 L aquaria filled with aerated and filtered artificial seawater with a salinity of 35 ppm. The fish were allowed to acclimatize for a period of 14 days at a normal water temperature of 20 °C and a natural photoperiod. Throughout the acclimatization period, the fish were fed with a commercial diet twice a day at 3% body weight. Water physicochemical parameters (dissolved oxygen, ammonia, nitrite, nitrate, pH, general hardness, carbonate hardness, and temperature) were monitored daily and kept within recommended levels to ensure good water quality.

### 2.2. Fish Bioassay and Sampling

Two experimental conditions were established: (1) the control group (0 μg/L PS-NPs), and (2) the group exposed to PS-NPs (100 μg/L PS-NPs). In each of the experimental conditions, 10 fish were randomly distributed in duplicated tanks per condition (20 L) with 5 fish per tank (N = 20), allowing a randomized distribution of biomass in the experimental conditions. Throughout the exposure period, the water quality parameters (as described for the acclimatization and maintenance period) were maintained within the recommended levels. Under the PS-NP experimental conditions, fish were exposed to PS-NPs (Bangs Laboratory Inc., Fishers, IN, USA) for 14 days; 50% of the medium was renewed every 2 days, and PS-NPs were added to each PS-NP treatment group in order to obtain a concentration of 100 μg/L. Fish were fed daily ad libitum with a commercial diet, the bottoms of the aquariums were cleaned to avoid the accumulation of nitrogenous wastes, and fish were observed for abnormal behavior.

Before sampling, the fish were fasted for 2 days to avoid the accumulation of food particles in the gut during sampling and the interaction of the food pellets with NPs. After 14 days of exposure, all fish were euthanized by anaesthetizing them in a tricaine methanesulfonate (MS-222, 1 g/L) bath. Fish were then weighed and measured, and heparin-coated needles and syringes were used to draw blood from the caudal vein of the fish. Immediately after collection, blood smears were made, and the remaining blood was placed in Eppendorf tubes (with heparin and kept on ice) for hematological analysis. Subsequently, the intestine, gills, and liver were excised, and the liver was weighed, rapidly frozen in liquid nitrogen, and kept at −80 °C until further analysis. Part of the gills and sections of different parts of the intestine were sampled and fixed in 10% formaldehyde for histological analysis. All experimental procedures were undertaken in compliance with the Animal Experimentation and Spanish regulations, following the International Guiding Principles for Biomedical Research Involving Animals (EU Directive 2010/63/EU) (license with reference CEEAH 4121-CEEA-UAB). To prevent or minimize accidental contamination by PS-NPs, we used glass aquaria, in addition to glass or metal equipment and silicone tubing. Experiments were conducted in closed clean rooms to reduce airborne contamination, or under laminar flow hoods. Solutions, water, and other reagents were filtered to remove plastic particles. Researchers used latex disposable gloves and 100% cotton laboratory coats to reduce the dispersion of airborne plastic fibers.

### 2.3. Fitness Indicators

Fish survival was 100% (N = 20/20) following the experimental exposures. At the end of the experiment, fish length (cm) was determined by measuring from the anterior end to the tip of the caudal fin. Additionally, the fish were weighed (g), and Fulton’s condition factor (K) was computed using the fishes’ weight and length. The liver was then sampled and weighed (g), and the hepatosomatic index (HSI) was calculated from these values following the formula described by Rizzo and Bazzoli [30] for the estimation of HSI and K:

HSI = (liver weight (g))/(total weight (g)) × 100

K = (weight (g))/(l [length (cm)] ^3) × 100

### 2.4. Hematological Profile

Blood samples were analyzed using an automated laser flow blood cell analyzer (Sysmex XN-1000 V, SYSMEX, Sant Just Desvern, Spain) with veterinary software version 3.04, with a betta version for bird blood analysis adapted for fish. Hematocrit, red cell count, leucocyte count, hemoglobin concentration, mean corpuscular cell volume, and thrombocyte count were determined. The panoptic fast staining of blood smear slides for the differential leucocyte count was performed. The number of nucleated cells (lymphocytes, monocytes, and heterophils) was counted by examining one hundred leukocytes in a smear.

### 2.5. Biochemical Analysis in Plasma

Cholesterol, triglycerides, alkaline phosphatase (ALP), aspartate aminotransferase (AST), and alanine transaminase (ALT) were determined in the plasma of fish using commercial kits (Olympus Systems Reagents; Olympus Life and Material Science Europe GmbH, Hamburg, Germany) and following the instructions described by the manufacturer. An automatic analyzer (Olympus Diagnostica, GmbH, Freiburg, Germany) was used to analyze all the parameters.

### 2.6. Histological Analysis

Gills and intestine samples were dehydrated in graded ethanol and cleaned in an organic solution (xylene) before being embedded in paraffin. The tissues were then processed using an automated modular paraffin embedding tissue processor (Especialiadades Médicas Myr, S.L, Valencia, Spain). Sections of 4 μm thickness were obtained using a microtome (LEICA RM2125 RTS, Leica Biosystems, Barcelona, Spain), and these were mounted on silane-coated slides. The slides were then stained with hematoxylin and eosin (H&E) (MYR CITOLAB) and examined under a light microscope (LEICA DM5000 B, Wetzlar, Germany).

### 2.7. Gene Expression Profiling

#### 2.7.1. RNA Extraction and Complementary DNA (cDNA)

Total RNA was extracted from the fish organs using 1 mL of Tri Reagent^®^ (Sigma-Aldrich, Saint Louis, MO, USA) and following the manufacturer’s instructions. First, the tissues were homogenized individually in 1 mL of Tri Reagent^®^ (Sigma-Aldrich) using a Polytron, and the homogenate was allowed to incubate for 5 min at room temperature. The Polytron was cleaned using diethylprocarbonate (DEPC) water between samples and with NaOH between experimental groups. Subsequently, 0.1 mL of 1-Bromo-3-chloropropane (BCP) was added to each sample, mixed vigorously using a vortex, and incubated for 10 min at room temperature.

Following the incubation, the samples were centrifuged for 15 min at 13,000× *g* and 4 °C. Then, the aqueous phase was transferred to a clean tube with 0.5 mL of 2-propanol (stored at −20 °C) and mixed softly with care to recover the RNA. The samples were then incubated for 10 min at room temperature and centrifuged for 10 min at 13,000× *g* and 4 °C. Finally, the RNA pellet was washed twice with 1 mL of ethanol at 75% (kept at 4 °C), air-dried for 10 min, dissolved in DEPC water using filter tips, and incubated at 56 °C for 10 min. Following extraction, RNA was quantified and assessed for purity using a NanoDrop Spectrophotometer (Thermo Fisher Scientific, Waltham, MA, USA). Then, 1 µg of total RNA was used to perform reverse transcription using the iScriptTM cDNA synthesis kit (BIO-RAD Laboratories, Hercules, CA, USA), following the manufacturer’s instructions.

#### 2.7.2. Real-Time Quantitative PCR (RT-qPCR)

The efficiency of amplification for each primer pair was determined using serial 5-fold dilutions of pooled cDNA. This was calculated using the formula E = 10 (−1/s), where ‘s’ represents the slope generated from the serial dilutions [31]. Reverse transcription–quantitative polymerase chain reaction (RT-qPCR) was performed on a Bio-Rad CFX384 Real-Time PCR Detection System with iTaqTM Universal SYBR^®^ Green Supermix (BIO-RAD Laboratories, Hercules, CA, USA), following the manufacturer’s instructions. Samples were tested in triplicate and the thermal cycle conditions were as follows: 1 cycle at 95 °C for 5 min, followed by 40 cycles at 85 °C for 30 s, and 72 °C for 30 s. Expression data obtained from three independent replicates were used to calculate the threshold cycle (Ct) value. After evaluating the efficiency of the primers, RT-qPCR analysis of each sample was performed following the previously outlined protocol.

#### 2.7.3. Normalization of Target Genes

The most appropriate housekeeping gene in the sample organ was evaluated using the NormFinder application, and the expression of the target genes was normalized based on the results. Potential reference genes were elongation factor 1 alpha (*ef1α*), beta-actin (*bact*), and glyceraldehyde-3-phosphate dehydrogenase (*gapdh*). The target genes were peroxisome proliferator-activated receptor alpha and beta (*pparα*, *pparβ*), leptin (*lep*), insulin-like growth factor 1 (*igf1*), glucocorticoid receptor (*gr1*), butyrylcholinesterase (*bche*), superoxide dismutase (*sod*), catalase (*cat*), glutathione peroxidase 1 (*gpx1*), and glutathione-S-transferase3 (*gst3*). The ΔΔCt method [31] was used to calculate the relative gene expression.

### 2.8. Data Analysis

Data analysis is presented as mean ± standard deviation (SD). The Shapiro–Wilk test was initially used to determine if the data were normally distributed. Data that followed normal distribution were further analyzed with the Welch correction *t*-test. Data that did not follow normal distribution were further analyzed with the Mann–Whitney *t*-test. Statistics were calculated using GraphPad Prism version 8.0 for Windows (GraphPad Software, Inc., Boston, MA, USA). Figures displaying an asterisk (*) indicate significant differences between the control group and the PS-NP-exposed group. A significance level of *p* < 0.05 was set for the statistical analysis.

## 3. Results

### 3.1. Biometric Analysis

**Figure 1:**
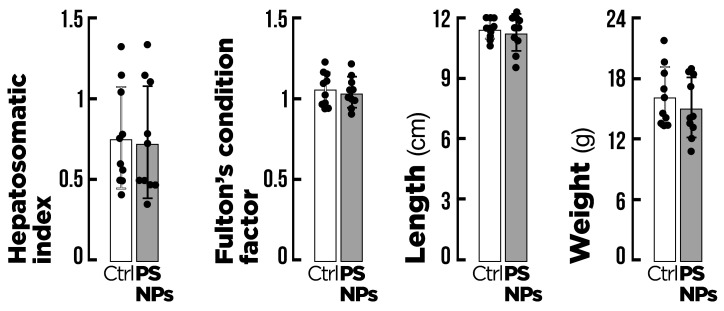
Biometric indicators of gilthead seabream (*Sparus aurata*) after exposure to the two experimental conditions for 14 days (n = 20). Ctrl = control group (0 μg/L PS-NPs 20 °C); PS-NPs = polystyrene nanoplastics group (100 μg/L PS-NPs at 20 °C). Values are presented as mean ± standard deviation, *p* < 0.05.

In the present study, the exposure of fish to PS-NPs did not affect the fitness indicators. There were no significant (*p* < 0.05) effects on body weight, length, condition factor, or hepatosomatic index for either the control or exposed fish (Figure 1).

### 3.2. Hematological Responses

**Figure 2:**
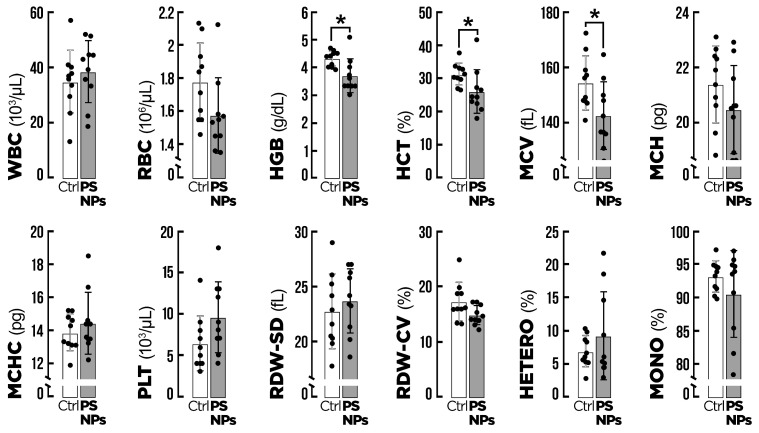
Hematological indicators of gilthead seabream (*Sparus aurata*) after exposure to the two experimental conditions for 14 days (n = 20). Ctrl = control group; PS-NPs = polystyrene nanoplastics group. Values are presented as mean ± standard deviation, *p* < 0.05. WBC = white blood cell count, RBC = red blood cell count, HGB = hemoglobin concentration, HCT = hematocrit, MCV = mean corpuscular cell volume, MCH = mean corpuscular hemoglobin, MCHC = mean corpuscular hemoglobin concentration, PLT = platelets, RDW-SD = red cell distribution width—standard deviation, RDW-CV = red cell distribution width—coefficient of distribution, HETERO = heterophils, MONO = monocytes. All data are expressed as mean ± standard deviation. * *p* < 0.05.

The effects of PS-NPs on the hematological indicators of the fish are presented in Figure 2. There were no significant effects on white blood cell count, red blood cell count, mean corpuscular hemoglobin, or platelets compared to the control group. Hemoglobin concentration reduced significantly (*p* = 0.005) in the PS-NP group (3.68 ± 0.62) compared to the control (4.31 ± 0.28). A significant decrease in hematocrit values was observed for the group exposed to PS-NPs (25.99 ± 6.71) compared to the control (31.26 ± 3.30). There was a significant reduction in mean corpuscular cell volume in the groups exposed to PS-NPs (142.50 ± 12.37) compared to the control (154.40 ± 9.88).

### 3.3. Biochemical Responses

**Figure 3:**
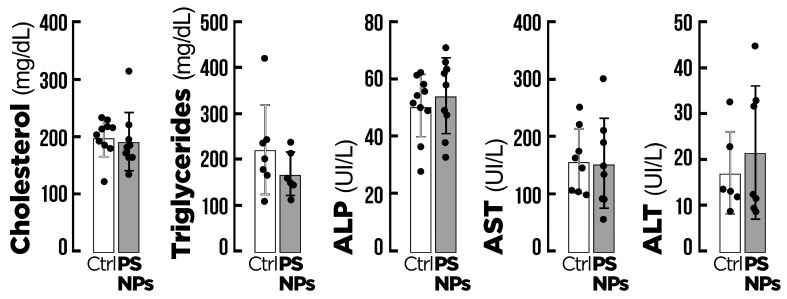
Biochemical indicators of gilthead seabream (*Sparus aurata*) after exposure to the two experimental conditions for 14 days (n = 20). Ctrl = control group; PS-NPs = polystyrene nanoplastics group. Values are presented as mean ± standard deviation, *p* < 0.05. ALP = alkaline phosphatase, AST = aspartate aminotransferase, ALT = alanine transaminase. All data are expressed as mean ± standard deviation.

The effects of PS-NPs on the biochemical indicators of the fish are presented in Figure 3. There were no significant effects on cholesterol, triglycerides, ALP, AST, or ALT.

### 3.4. Histological Changes

**Figure 4:**
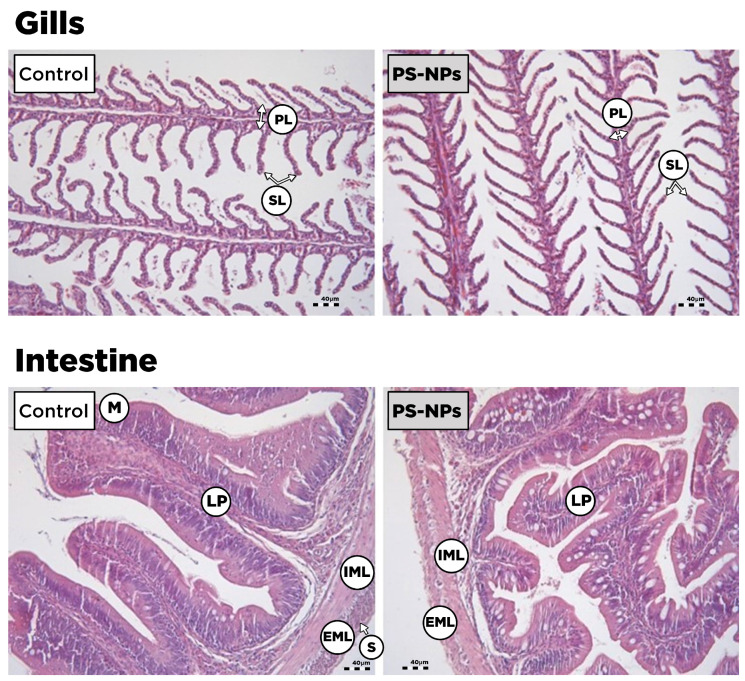
Photomicrographs of *S. aurata* gills and intestine after exposure to the two experimental conditions for 14 days (n = 5). In gills, we observed normal primary lamella (PL) and secondary lamella (SL) in both the control and PS-NP groups. In the intestine, we observed a normal mucosa layer (M), lamina propria (LP), serosa (S), and muscularis mucosa [internal muscle layer (IML) and external muscle layer (EML)] in both the control and PS-NP groups.

The results of the histology of fish gills after exposure to the experimental conditions are presented in Figure 4. In the control group, the gills exhibited a normal appearance with no obvious signs of cellular damage, excessive mucous, or inflammation. The primary lamella (gill filament) and the cartilaginous skeleton that supports the secondary lamella showed a normal morphology. The secondary lamellae were clear, appeared as finger-like filaments, and were arranged nearly at a right angle to the primary lamella. The secondary lamella appeared highly vascularized with erythrocytes within the capillary lumen. The outer layer of the secondary lamella showed normal epithelial cells. After exposure to PS-NPs, the gills showed similar results to the control group.

In terms of the intestine, the control group showed normal morphology for all intestinal layers. The mucosa consisted of the mucosal layer lined with goblet cells. The lamina propria revealed normal morphology with lymphocyte cells and connective tissues. The muscularis mucosa includes the internal and external muscle layers. After exposure to PS-NPs, the histological anatomy of the exposed fish intestine showed no noticeable differences compared to the control.

### 3.5. Molecular Analysis

The PCR amplification efficiencies of each gene ranged from 92.5% to 105.7% (Table 1). The efficiency values were estimated by standard curves generated using 5-fold dilutions of pooled cDNA. Before the analysis of the target genes, the variation in each reference gene in the liver was assessed. The stability values of the candidate reference genes (based on the NormFinder calculations) were 0.028 for *ef1*, 0.030 for *gadph*, and 0.041 for *bact*. Hence, the most stable reference gene in the present study was *ef1*.

**Figure 5:**
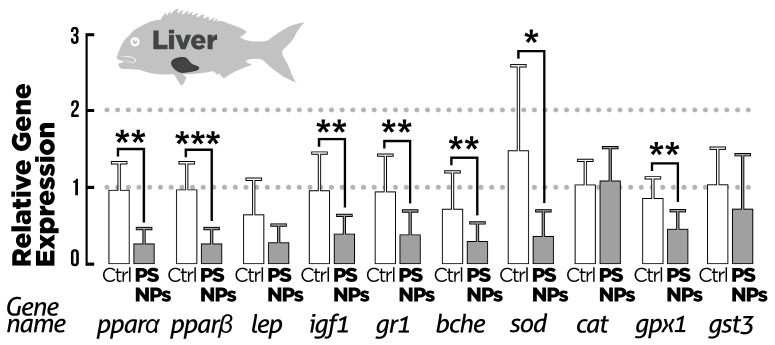
Gene expression of target genes in the liver of *S. aurata* after exposure to the two experimental conditions for 14 days (n = 20). Ctrl = control group; PS-NPs = polystyrene nanoplastics group. Values are presented as mean ± standard deviation, *p* < 0.05. All data are expressed as mean ± standard deviation. * *p* < 0.05, ** *p* < 0.01, *** *p* < 0.001. Analyzed genes were peroxisome proliferator-activated receptor alpha and beta (*pparα*, *pparβ*), leptin (*lep*), insulin-like growth factor 1 (*igf1*), glucocorticoid receptor (*gr1*), butyrylcholinesterase (*bche*), superoxide dismutase (*sod*), catalase (*cat*), glutathione peroxidase 1 (gpx1), and glutathione-S-transferase 3 (*gst3*).

The mRNA levels (Figure 5) for lipid metabolism-related genes (*pparα*, *pparβ*, *lep*) showed varied responses after the exposure conditions. There was a significant decrease in *pparα* and *pparβ* transcriptional levels in the PS-NPs group compared to the control. For *lep* transcriptional levels, no significant effect was observed after exposure to PS-NPs compared to the control. The transcriptional level for genes related to growth and development (*igf1*) was significantly decreased after exposure to the PS-NPs. Concerning the expression of the stress-related gene (*gr1*), there was a significant decrease in transcripts after exposure to the PS-NPs. The transcriptional levels of genes involved in detoxification (*bche*) showed a significant decrease in the PS-NP group in comparison to the control. For antioxidant-related genes, the mRNA levels of *sod* and *gpx1* significantly decreased after exposure to the PS-NPs, while *cat* transcriptional levels were unaffected. The transcriptional levels of *gst3* showed no significant effect after exposure to PS-NPs.

## 4. Discussion

The present results indicate that the juveniles of *S. aurata* enduring a sub-chronic (14 days) exposure to 100 μg/L of polystyrene nanoplastics did not show major changes in terms of the hematological and metabolic plasmatic parameters measured at the end of the exposure period. Likewise, we did not observe histopathological alterations in the structure of the gills and intestine. Even if mild downregulation of the gene transcripts involved in lipid metabolism and oxidative stress was observed in the liver, the growth and condition indexes remained unaffected by PS-NP exposure.

Blood analysis is a reliable, useful, and cost-effective method for evaluating animal health that is used to calibrate the health of fish [32] exposed to plastic contaminants. However, there is still limited literature on fish hematological changes after exposure to nanoplastics, and the few studies available have focused mainly on the effects of microplastics (MPs) on fish hematology, with significant changes observed in some of the parameters [33,34,35,36,37]. For instance, Hamed et al. (2019) reported that some physiological effects included alterations in the red blood cells, hemoglobin, hematocrit, and white blood cells of Nile tilapia (*Oreochromis niloticus*) after waterborne exposure to 50 μm polyethylene MP for 15 days [33]. However, NPs and MPs differ in their bioavailability, uptake, and transport properties, as well as the way they affect hematological parameters [38]. Our data indicate that in *S. aurata* juveniles, the main hematological parameters measured did not change, except for a mild reduction in hemoglobin concentration, hematocrit, and mean corpuscular cell volume, as has also been described in *O. niloticus* for medium-term exposure to nanoplastics [39]. The changes in hematological parameters induced by NP exposure may be interpreted as a telltale for incipient inflammatory states or fully formed immune responses, and could be linked to oxidative stress, impaired hematopoiesis, or the adsorption of hemoglobin [40,41]. Previous studies have documented that PS-NPs can induce oxidative stress in addition to pro-inflammatory responses and the destruction of red blood cells, thus lowering MCV in fish [42]. However, we did not observe changes in the plasmatic levels of seabream’s white blood cells, heterophils, or monocytes, the main culprits for cell-mediated inflammatory responses [43]. In this sense, our results agree with those of Brandts et al. (2022), who did not observe changes in hematological parameters in *Carassius auratus* after 30 days of waterborne exposure to 100 μg/L PS-NP [22].

Monitoring metabolic-related biochemical parameters provides valuable information on the general well-being of fish, and how different factors such as environmental fluctuations and pollution can affect their health status. Here, we have analyzed the plasmatic levels of cholesterol, triglycerides, alkaline phosphatase (ALP), alanine aminotransferase (ALT), and aspartate aminotransferase (AST), which did not change at day 14 of PS-NP exposure compared to the controls. The absence of changes in plasmatic metabolic markers was also described for *Dicentrarchus labrax* after short-term exposure to up to 10 mg/L of polymethylmethacrylate NP [44]. However, other studies have described higher triglyceride levels in juvenile *Siniperca chuatsi* exposed to nanoplastics (5.14 μg/L) when compared to the control group [45]. In addition, a significant increase in triglyceride levels was found for adult zebrafish (*Danio rerio*) co-exposed to microplastic and cypermethrin, and no changes were observed for the individual groups [46]. Clearly, a marked species-specific pattern of short-to-medium-term effects of NPs on lipid metabolism can be drawn from the published results. In fish, as in mammals, alkaline phosphatases form a distributed system that modulates the inflammatory onsets related to the adsorption or ingestion of plastic fragments, xenobiotics, and pathogens, and regulates metabolic changes [47,48,49,50]. In this sense, the absence of changes observed in plasmatic levels of ALP precludes an inflammatory state 14 days after exposure to 0.1 mg/L PS-NPS in *S. aurata* juveniles. Plasma ALT and AST levels rise in fish exposed to contaminants and metabolic imbalances that may lead to cellular damage [51,52,53], and our results suggest an unaltered regulation of whole-organism transit and metabolism of lipids in seabream exposed sub-chronically to PS-NPs. In this regard, the expression of hepatic transcripts related to lipid metabolism and peroxisome proliferator-activated receptor alpha and beta (*pparα* and *pparβ*) decreased slightly, whereas the expression of transcripts for letpin (*lep*) remained unchanged at day 14 post-exposure.

The liver is a multifunctional organ involved in detoxification processes, playing a central role through metabolism and biotransformation. Both PPARα and β are involved in the regulation of fatty acid absorption, adipogenesis, the modulation of autophagy and apoptotic responses in hepatocytes [54], and the regulation of anti-inflammatory responses [55]. A diminished expression of *pparα*/*β* transcripts, even if slight, as observed in seabream exposed to PS-NPs, may imply a lesser functionality of inflammation, glucose homeostasis, lipid metabolism, and the regeneration of hepatocytes [56]. However, the absence of changes in the plasma levels of cholesterol and triglycerides, together with the unaltered expression levels of leptin, indicate that the impact on lipid metabolism was minimal. In fact, changing levels of leptin act as an adiposity marker that alerts of metabolic disorders, usually related to alterations in food intake [57], that may translate to growth impairment. As mentioned before, we did not observe changes in growth rates or somatic indexes (Fulton’s condition factor and hepatosomatic index (HSI)), which are regarded as indicators of altered lipid homeostasis related to growth, in fish exposed to PS-NPs at day 14. However, we cannot rule out an effect on growth or food assimilation rates if the exposure to NPs become chronic, as suggested by the downregulation of insulin-like growth factor 1 (*igf1*) transcripts and stress-related glucocorticoid receptor (*gr1*) transcripts. The *Igf1* gene, which is synthesized primarily in the liver, is directly involved in promoting tissue growth, and as such, constitutes a marker of somatic growth through the activation of the (GH)/insulin-like growth factor (IGF) axis [58]. An altered regulation of GH/IGF growth signaling in stressful environments has been described to affect fish performance across generations in medaka (*Oryzias latipes*) exposed to plastic contaminants [59]. In zebrafish, exposure to 0.5 mg/L of PS-NP disrupted PPAR signaling, inducing dysgenesis, developmental alterations, and lipid transport dysregulation [60]. In yellow croaker (*Larimichthys crocea*), a 21-day feeding diet enriched with PS-NPs impaired the survival and growth of fish in a dose-dependent manner, eliciting oxidative stress [61]. Taking all together, these results suggest that the noxious effects on developmental and growth rates in fish chronically exposed to nanoplastics may be enhanced following a previous and sustained sub-chronical exposure, even at the concentrations used in our study. However, an accurate description of the effect of MP-NPs on fish growth is still missing.

We also observed a variable pattern of gene expression levels concerning oxidative stress. Superoxide dismutase (*sod*) and glutathione peroxidase 1 (*gpx1*), but not catalase (cat) or glutathione-S-transferase3 (*gst3*) mRNA expression levels in the fish liver, showed a slight but significant decrease in the PS-NP group when compared to the control. This suggests a possible decline in the capacity to protect cells against oxidative stress. However, the antioxidant response in fish to NP exposure is highly variable in terms of the species considered and the enzymes involved. Previous studies have reported an upregulation [44,62] and downregulation [63] of *sod* mRNA levels in fish after exposure to NPs. Some studies have also linked the downregulation of the *sod* gene to a decreased ability to fight pathogens and infections in fish [64,65]. Considering that fish depend on innate immunity in the early development stage, reduced *sod* mRNA levels can cause long-term immunity and adaptation to multiple stressors in the external environment. Fish exposed to PS-NPs revealed a significant decrease in liver *gpx1* mRNA levels. Regarding fish exposure to nanoplastics, an upregulation has been documented in zebrafish liver after 21 days of exposure to 50 nm of PS-NP at 0.07 mg/L [66]. Overall, a downregulation of antioxidant-related transcripts may indicate an exhaustion of antioxidant enzymes at the end of the 14-day exposure, or, alternatively, alterations in antioxidant signaling in seabream exposed to PS-NPs, which, as discussed before, may exert an effect of growth rates if the exposure is prolonged. The same may be true concerning the downregulation of a marker of xenobiotic metabolism, the transcript for butyrylcholinesterase (*bche*). The *bche* gene is involved in the metabolism of some xenobiotics and neurotransmitters [67], acting as a bioscavenger to regulate detoxification [68]. This implies that the decrease in liver *bche* mRNA levels observed in this study can reduce the ability of fish to detoxify toxic compounds. As the *bche* gene is also an important component of the neurotransmission processes, this therefore suggests possible alterations in neurophysiological activities in fish during chronic exposure to nanoplastics.

In line with the results discussed above, we did not observe histopathological alterations in the gills or the intestine lining. The gills exhibited a normal appearance with no obvious signs of cellular damage, excessive mucous, or inflammation in the control and PS-NP exposure conditions. In the intestine, the histological anatomy of the fish showed no noticeable differences under the PS-NP exposure conditions compared to the control. The lack of histological alterations in the present study agrees with a previous study where polystyrene microplastics (80 nm and 8 μm), even at high concentrations (5 mg/L), did not cause visible alterations in the intestinal morphology of Largemouth bass (*Micropterus salmoides*), Grass carp (*Ctenoppharyngodon idella*), or Jian carp (*Cyprinus carpio* var. Jian) [69]. According to the authors, this could be attributed to the ability of fish to promote self-repair through the upregulation of some inflammatory factors when exposed to low concentrations of nanoplastics.

## 5. Conclusions

In conclusion, the present study demonstrated that 14 days of exposure of *S. aurata* juveniles to PS-NPs did not affect fitness indicators, but led to mild alterations in hematological and biochemical parameters. The histological anatomy of the gills and intestine showed no noticeable differences compared with the control. After experimental exposure, the mRNA expression genes related to lipid metabolism (*pparα*, *pparβ*), growth and development (*igf1*), detoxification (*bche*), and oxidative stress (*sod*, *gpx1*) were significantly downregulated, indicating the activation of different response mechanisms in the studied fish. Overall, gilthead seabream seems to respond to medium-term exposure to PS-NPs with slightly impaired lipid metabolism and altered antioxidant capacity, which will probably affect growth performance if the exposure is maintained and becomes chronic in stressful environments.

## Figures and Tables

**Table 1:** Sequences and efficiency of primers used for quantitative real-time PCR in the liver of *S. aurata*.

Gene Name	Acronym	Accession No.	Forward	Reverse	Efficiency (%)
Elongation factor-1 α	*ef1*	AF184170	CCCGCCTCTGTTGCCTTCG	CAGCAGTGTGGTTCCGTTAGC	101.0
Beta-actin	*bact*	X89920	TCCTGCGGAATCCATGAGA	GACGTCGCACTTCATGATGCT	101.9
Glyceraldehyde-3-phosphate dehydrogenase	*gadph*	DQ641630	TGCCCAGTACGTTGTTGAGTCCAC	CAGACCCTCAATGATGCCGAAGTT	100.6
Peroxisome proliferator-activated receptor Alpha	*pparα*	AY590299	GCAGCCTGTGAGTCTTGTGAGTGA	CTCCATCAGGTCTCCACACAGC	98.2
Peroxisome proliferator-activated receptor Beta	*pparβ*	AY590301	CGTGTTCGGGATTCGGGACT	CACCCTGTCGTGCTGCTCTGTA	105.7
Leptin	*lep*	MG570179	CAGCCTGATCTCAGACGACCTTGACAAC	TGATCCAGGAATCCAGACAGCGAAGA	92.5
Insulin-like growth factor I	*igf1*	AY996779	GCCACACCCTCTCACTACTG	AAGCAGCACTCGTCCACA	96.2
Transforming growth factor 1 beta	*tgf1b*	P01137	GCATGTGGCAGAGATGAAGA	TTCAGCATGATACGGCAGAG	93.2
Butyrylcholinesterase	*bche*	B013682	CAGGTACTCCCAACACGGTG	ATCTCGTAGCCGTGCATGAC	97.7
Glucocorticoid receptor	*gr1*	AJ937873	TGTTCAGCCACCCACCCATCGG	GCGTGATACATCGGAGTGAATGAAGTCTTG	100.9
Superoxide dismutase	*sod*	JQ308833	CCTGACCTGACCTACGACTATGG	AGTGCCTCCTGATATTTCTCCTCTG	99.9
Catalase	*cat*	JQ308823	TGGTCGAGAACTTGAAGGCTGTC	AGGACGCAGAAATGGCAGAGG	102.7
Glutathione peroxidase 1	*gpx1*	DQ524992	GAAGGTGGATGTGAATGGAAAAGATG	CTGACGGGACTCCAAATGATGG	99.0
Glutathione-S-transferase3	*gst3*	JQ308828	CCAGATGATCAGTACGTGAAGACCGTC	CTGCTGATGTGAGGAATGTACCGTAAC	105.5

## Data Availability

The original contributions presented in this study are included in the article. Further inquiries can be directed to the corresponding author(s).
